# Virological characterization of influenza H1N1pdm09 in Vietnam, 2010-2013

**DOI:** 10.1111/irv.12323

**Published:** 2015-06-09

**Authors:** Hang K L Nguyen, Phuong T K Nguyen, Thach C Nguyen, Phuong V M Hoang, Thanh T Le, Cuong D Vuong, Anh P Nguyen, Loan T T Tran, Binh G Nguyen, Mai Q Lê

**Affiliations:** a National Institute of Hygiene and EpidemiologyHanoi, Vietnam; b 108 Military Central HospitalHanoi, Vietnam; c National Hospital of Traditional MedicineHanoi, Vietnam; d Bach Mai HospitalHanoi, Vietnam

**Keywords:** Influenza A/H1N1pdm09, the vaccine virus A/California/07/2009, Vietnam, virological characterization

## Abstract

**Objectives:**

Influenza A/H1N1pdm09 virus was first detected in Vietnam on May 31, 2009, and continues to circulate in Vietnam as a seasonal influenza virus. This study has monitored genotypic and phenotypic changes in this group of viruses during 2010–2013 period.

**Design and setting:**

We sequenced hemagglutinin (HA) and neuraminidase (NA) genes from representative influenza A/H1N1pdm09 and compared with vaccine strain A/California/07/09 and other contemporary isolates from neighboring countries. Hemagglutination inhibition (HI) and neuraminidase inhibition (NAI) assays also were performed on these isolates.

**Sample:**

Representative influenza A/H1N1pdm09 isolates (*n *=* *61) from ILI and SARI surveillances in northern Vietnam between 2010 and 2013.

**Main outcome measures and results:**

The HA and NA phylogenies revealed six and seven groups, respectively. Five isolates (8·2%) had substitutions G155E and N156K in the HA, which were associated with reduced HI titers by antiserum raised against the vaccine virus A/California/07/2009. One isolate from 2011 and one isolate from 2013 had a predicted H275Y substitution in the neuraminidase molecule, which was associated with reduced susceptibility to oseltamivir in a NAI assay. We also identified a D222N change in the HA of a virus isolated from a fatal case in 2013.

**Conclusions:**

Significant genotypic and phenotypic changes in A/ H1N1pdm09 influenza viruses were detected by the National Influenza Surveillance System (NISS) in Vietnam between 2010 and 2013 highlighting the value of this system to Vietnam and to the region. Sustained NISS and continued virological monitoring of seasonal influenza viruses are required for vaccine policy development in Vietnam. 3

## Introduction

Influenza A/H1N1pdm09 was detected first in March 2009 in Mexico which then spread globally, resulting in a pandemic with more than 18 000 laboratory-confirmed deaths in more than 200 countries.^1^ Similar to previous pandemic influenza viruses, influenza A/H1N1pdm09 virus has become endemic, and it is cocirculating with influenza A/H3N2 and B viruses during seasonal epidemics.

**Figure 1:**
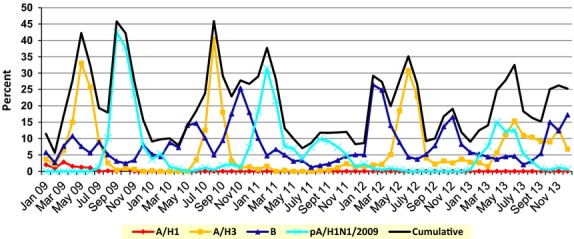
Proportion of ILI patients in Vietnam infected with (RT-PCR reactive) the influenza virus genotypes shown between 2009 and 2013.

Vietnam was the 54th country to identify cases of infection with influenza A/H1N1pdm09. The first case was detected on May 31, 2009, and then spread rapidly in July 2009; by August–September, 85–90 percent of influenza infection in patients in Vietnam was A/H1N1pdm09.^2^ Global modeling estimated up to 284 000 deaths in the first 12 months of A/H1N1pdm09 transmission^3^; in Vietnam, according to the Ministry of Health, a total of 11 047 cases with 50 deaths were reported by December 21, 2009. During 2010–2013 period, 46·6% of total confirmed influenza cases were determined to be A/H1N1pdm09 in 2009, and then, it decreased to 28% by 2010, when it cocirculated with A/H3N2 and B viruses. In 2011, 74·1% of reported influenza cases were for A/H1N1pdm09. After a lower circulation in 2012 of the predominant A/H3N2 viruses, the A/H1N1pdm09 viruses were detected in about 30% of cases in 2013 4 (Figure1).

Since the emergence of A/H1N1pdm09, molecular analysis has identified a number of mutations associated with variations in the clinical manifestations, pathogenesis, and immune response. For example, the amino acid substitution at position D222G/E/N (H1 numbering) in the HA molecule was associated with increased viremia, severe pneumonia, and deaths^2^,^5^,^6^; a substitution (I219K) in the glycan receptor-binding site of HA quantitatively increased its human receptor-binding affinity^7^; and substitutions D131E, S186, and A198E contribute to virulence in mice.^7^ In addition, substitutions I117V, I223R, and H275Y on the NA protein conferred reduced susceptibility to oseltamivir.^8^–^10^ Amino acid substitution in NP (D101G), PA L295P), and PB2 (E158E/A) was associated with virulence or enhanced transcription and replication activity.^7^ Furthermore, the potential to reassort with other seasonal influenza viruses may result in increased pathogenicity of influenza A/H1N1pdm09.^11^ For these reasons, WHO's Global Influenza Surveillance and Response System (GISRS) has requested countries to monitor influenza A/H1N1pdm09 viruses for important genetic, antigenic, or functional changes.

We have undertaken surveillance of influenza A/H1N1pdm09 in Vietnam in the post-pandemic period (2010–2013) with the objective of identifying mutations in the hemagglutinin (HA) and neuraminidase (NA) genes that may result in changes in severity of disease or alterations in the effectiveness of antiviral therapies and vaccines.

## Materials and methods

### Ethical approval

The study was conducted as part of the Ministry of Health-approved influenza surveillance system using samples taken from patients presenting to healthcare facilities with an influenza-like illness (ILI). The National Institute of Hygiene and Epidemiology (NIHE), Vietnam, provided ethical committee approval for the study. All participants provided written informed consent.

### Source of samples

All throat swabs were collected as part of the sentinel surveillance system for ILI and severe acute respiratory infection (SARI) in northern Vietnam between 2010 and 2013. The swabs were screened using standard methodology (conventional reverse transcriptase-polymerase chain reaction, RT-PCR) employing specific primer sets targeted to the matrix (M) and hemagglutinin genes (HA) at the National Influenza Centre of the National Institute of Hygiene and Epidemiology (NIC-NIHE) in Hanoi. Influenza isolates were then cultured from throat swabs found to contain influenza A or B viruses by RT-PCR. In addition, influenza A/H1N1pdm isolates were obtained from the influenza regional laboratories of the National Influenza Surveillance System (NISS), that is, the Pasteur Institute of Ho Chi Minh city (IP Ho Chi Minh), Pasteur Institute Nha Trang (IP Nha Trang), and Tay Nguyen Institute of Hygiene and Epidemiology (TIHE).^12^,^13^

Madin–Darby canine kidney cells (MDCK; American Type Culture Collection) were used to culture viruses. Viruses were stored at −80°C prior to analysis. Representative influenza A/H1N1pdm09 isolates with a minimum of eight hemagglutination units were selected for sequencing and neuraminidase inhibition assay at NIHE, Hanoi (Table1).

**Table 1:** : Sample size and mutation established during 2010–2013. Significant mutations in HA gene: G155E and N156K reduced HI titer, and D222N associated with increased virulence or severe and fatal disease

Amino acid mutation compared to A/California/07/2009						
Gene	Group/Subgroup	Number of virus (*n*)				Uncommon substitutions
		2010	2011	2012	2013	
HA common substitutions P83S, S203T, R223Q, I321V	2	1				
	3	9				
	4	9				
	6A				7	N38D, D97N, H138R, S185T, V249L, E374K, S451N
	6C		1		23	D97N, G155E, N156K, S185T, I216M, D222N, V234I, T241I, K283E, E374K, S451N, E499K
	7		6	4	1	D97N, S143G, S185T, A197T, N260D, E374K, S451N, E499K
	Total	61				
NA common substitution N248D	2	1				
	3	15				K84R, V106I
	5				5	V83L, V106I, I122V, V241I, I321V, N369K, N386S, D416N
	6B				5	N44S, V106I, N200S, V241I, N369K
	6C				10	N44S, V106I, V241I, N369K, N200S
	7A		3	1	1	G41R, N44S, V106I, V241I, N369K, Q313R
	7B		1	1	3	N44S, V106I, V241I, N369K
	Total	46				

### Hemagglutination inhibition (HI) assay

The hemagglutination inhibition assay was performed according to WHO protocols using reference antisera and antigen (A/California/7/2009 (H1N1-like) supplied by WHO (CDC, Atlanta, USA). The serotype of all influenza A/H1N1pdm09 isolates was reconfirmed by hemagglutination inhibition (HI) assay. The reference antisera and negative reference serum were treated with receptor-destroying enzyme (RDE; Denka Seiken, Tokyo, Japan), heat-inactivated for 30 min at 56°C, and then adsorbed against packed appropriate erythrocytes. HI assays were performed in V-bottom 96-well microtiter plates with 0·5% turkey RBC controls and negative controls. The reference serum was tested at an initial dilution of 1:10 and then at twofold serial dilutions to a maximum dilution of 1:1280. The HI titer was read as the reciprocal of the highest serum dilution causing complete inhibition of agglutination.

### Molecular characterization

#### RNA extraction and Polymerase Chain Reaction (PCR)

RNA extraction was conducted on a 140-μl aliquot of each isolate using a Viral RNA Extraction Kit (Qiagen, USA) according to the manufacturer's instructions. The RNA was transcribed to cDNA using the influenza A virus universal primer (Uni 12) AGC AAA AGC AGG as described^16^. The hemagglutinin (HA) and neuraminidase (NA) genes were amplified with segment-specific primer using HotStar HiFidelity Polymerase Kit (Qiagen, 202602).

#### Nucleotide sequencing and phylogenetic analysis

The PCR products were purified using a PCR purification kit (Qiagen, USA) and labeled with a BigDye Terminator v3·1 Cycle Sequencing Kit (Applies Biosystems, USA) according to the manufacturer's instructions and then analyzed on an ABI 3130 automatic DNA sequencer. Nucleotide sequences were assembled using DNASTAR v.8.0, and multiple sequence alignment using H1 numbering was conducted with CLUSTAL X for the major coding regions of HA and NA segments. The sources of sequences in phylogenetic analyses are shown in Figures 3 and 4. Phylogenetic trees were constructed by the maximum likelihood (ML) method with bootstrap-supported values. To quantify the amino acid sequence diversity of each lineage, we used MEGA 5 software package. To find the closest relative, sequences were compared with those in the NCBI database using BLAST.

### Neuraminidase inhibition assay

The active form of oseltamivir carboxylate (GS4071) was provided by Roche Laboratories, Inc., Basel, Switzerland. The reference influenza viruses A/Perth/265/2009 (wild-type virus) and A/Perth/261/2009 (resistant virus) were provided by the World Health Organization (WHO) Collaborating Center for Reference and Research on Influenza, Melbourne, Australia. The NAI assay was performed as described previously.^10^,^14^

## Results

The first wave of influenza A/H1N1pdm09 transmission in Vietnam lasted from the initial introduction on May 31, 2009, until early 2010, and a second wave of transmission occurred from November 2010 to April 2011, with a major peak in January 2011. While only occasional isolates of A/H1N1pdm09 were made in 2012, there was a further large outbreak of A/H1N1pdm09 infection beginning in January 2013 (Figure1).^15^ Data from NISS in Vietnam 2009–2013 indicated year-round circulation of seasonal influenza viruses with frequent cocirculation of influenza A/H1N1pdm09, A/H3N2, and influenza B (Figure1) throughout the 4-year period.

**Figure 2:**
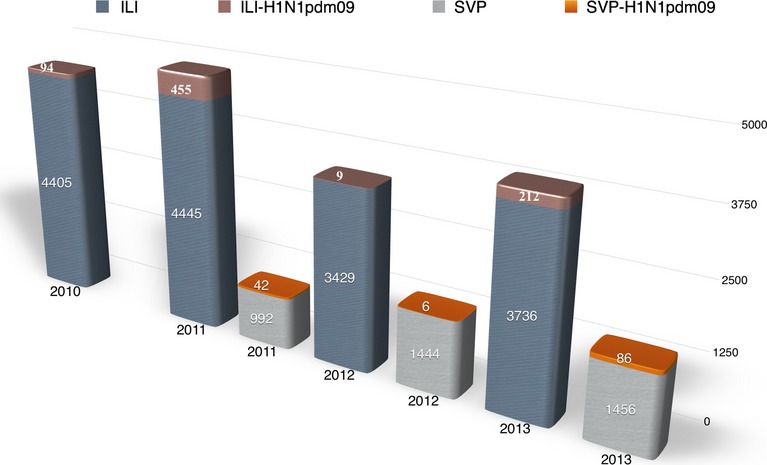
Number of A/H1N1pdm09 infections detected by RT-PCR during ILI and SVP surveillance, in Vietnam, 2010–2013.

Using RT-PCR, the range of annual influenza A-positive specimens was determined to be from 8·0% (2012) to 13·8% (2013), and a total of 770 influenza A/H1Npdm09-confirmed cases were detected from 16017 samples collected from ILI surveillance during 2010–2013 (4·8%) (Figure2). An additional 134 A/H1N1pdm09-confirmed cases were recognized from 3892 samples (3·44%) collected through SARI surveillance from 2011 to 2013 (Figure2). Swabs from patients identified by RT-PCR were inoculated on to MDCK cells, and 61 representative influenza A/H1N1pdm09 isolates with an HI titer of 80 or greater were selected for this study (Table1, Table2).

**Table 2:** Hemagglutination-inhibition reactions of studied influenza A/H1N1pdm09

Antigen testing	Reference antisera A/California/07/2009	Group/subgroup (*n*)						%(*n/N*)	Sequence change	Year (*n*)
A/California/07/2009	1280	2	3	4	6C	6A	7	61		
Studied viruses (*N* = 61)	1280	1	7	5	17	3	8	67·2		
	640		2	4	2	4	3	24·6		
	320				5			8·2	G155E and N156K	2011(1) 2013(4)

### Phylogeny of HA genes

**Figure 3:**
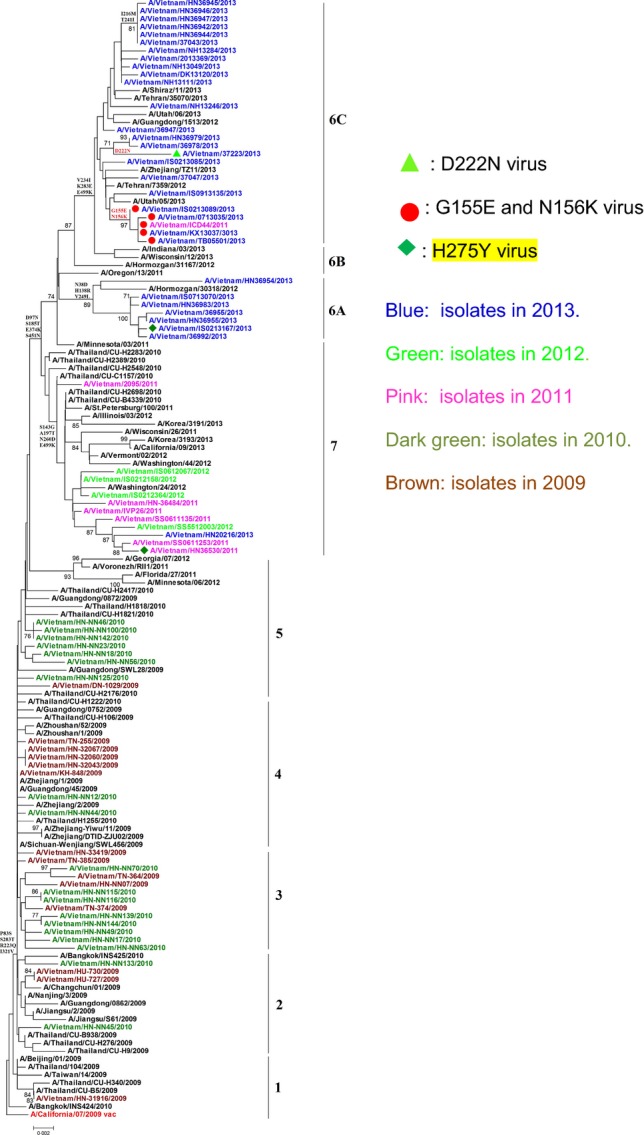
Maximum likelihood phylogeny of the HA genes of A/H1N1pdm09 viruses circulating in Vietnam, 2009–2013.

During the 4 years of circulation, the HA phylogeny of H1N1pdm09 in Vietnam has revealed nine genetic groups by maximum likelihood of 1000 bootstrap replicates to provide statistical support for each group generated.^16^ The four main groups 3, 4, 6, and 7 are designated in Figure3, Table1. The HA phylogenetic tree was developed from 61 studied isolates along with the HA gene of the vaccine candidate strain A/California/07/2009 and other isolates from Thailand, China, and Taiwan. Most isolates from 2010 diverged into group 3 (9 isolates) and group 4 (9 isolates) and clustered with isolates from Thailand and China; at the same time, they all shared amino acid changes P83S, S203T, R223Q, and I321V compared with A/California/7/2009 strain (Figure3, Table1). A total of 31 isolates from 2013 and an isolate from 2011 fell into group 6 with those from China, Teheran, and the USA: They were divided into two subgroups 6A and 6C (Figure3). Subgroup 6C covered most study isolates (24/31 isolates). Beside common amino acid changes of group 6 (D97N, S185T, S203T, E374K, and S541N), study viruses in subgroup 6C also showed substitutions at I216M, V234I, T241I, K283E, and E499K compared to the reference virus A/California/7/09.^16^,^5^ Additional significant changes found in Vietnamese isolates in group 6C included D222N (1 isolate) and G155E and N156K (5 isolates); notably, these mutations have previously been associated with increased virulence or severe and fatal disease (D222G/N) 17 or reduced HI titers when using ferret antiserum raised against A/California/7/2009 (G155E and N156K) (Figure3, Table2).^18^

Group 7 comprised eleven isolates and shared three additional amino acid changes at S143G, A197T, and N260D in comparison with group 6; the amino acid substitution E449K located at the root of clade 7 was first identified in 2011 and was also found in clade 6C. Subsequently, the substitutions P83S, S203T, R223Q, and I321V were found in all Vietnamese viruses (Table1). The HA phylogenetic tree also showed an isolate from 2010 that fell in group 2 viruses and was closely related to A/California/07/2009 and other viruses collected in the early pandemic period from Thailand, Taiwan, and China (Figure3).

### Phylogeny of NA gene

**Figure 4:**
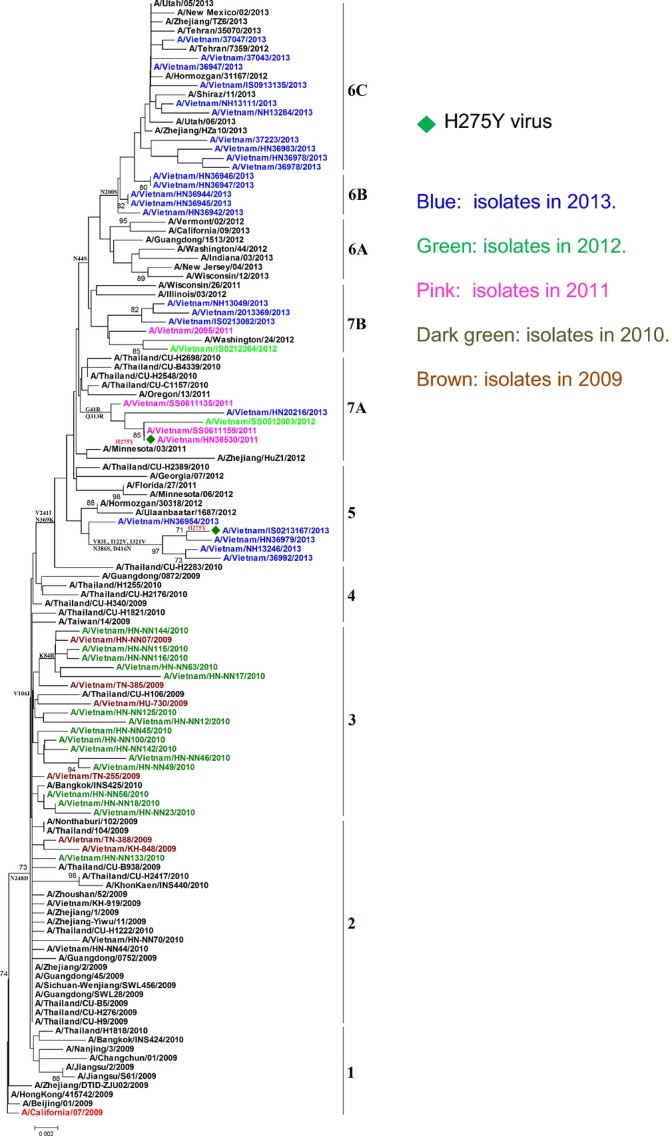
Maximum Likelihood phylogeny of NA genes of A/H1N1pdm09 viruses circulating in Vietnam, 2009–2013.

Phylogenetic analysis conducted on the NA gene of 46 of 61 virus isolates from 2010 to 2013 indicated that Vietnamese A/H1N1pdm09 isolates fell into seven groups/subgroups, with the dominant groups being group 3 (15 isolates) and subgroup 6C (10 isolates) (Table1; Figure4). The isolates collected in 2013 are predominant in this study (24/46) and concentrated in three groups/subgroups (5; and 6B, 6C) with common changes of N44S, V106I, and N248D, and more frequent in group 6 compared to A/California/07/2009 strain. A significant mutation at H275Y related to reduced susceptibility to oseltamivir was found in two isolates in 2011 and 2013 located in groups 7A and 5 (Figure4). Similar to the HA phylogenetic tree, the early isolates in 2010 were located in groups 2 and 3 with other viruses from neighboring countries. Group 3 was divided by a substitution of K84R (Figure4, Table1). Group 7 (7A and 7B) contains viruses collected during the years 2011, 2012, and 2013 that diverged by the changes of G41R and Q313R, and which were close with viruses from Thailand, USA, and China in the same period compared to group 6 (Figure4, Table1).

### Functional assay results

Following molecular tests, we evaluated the effect of mutations in the HA and NA genes on the biological function of influenza A/H1N1pdm09 by the hemagglutination inhibition (HI) assay and neuraminidase inhibition (NAI) assay. Almost all Vietnamese isolates (56 of 61, 92%) were inhibited by ferret antisera raised against A/California/07/2009-like reference viruses with similar HI titer of >640–1280 (Table2). However, a small proportion (8·2%) of viruses showed reduced reactivity by HI (titer of 320); each of these five viruses had amino acid substitutions G155E and N156K in the HA (four isolates from 2013 and one isolate from 2011). We cannot rule out the possibility that these mutations reflect passage effects in cell culture (Table2) as the original specimens were not directly sequenced.

The neuraminidase inhibition test was preformed in 46 clinical isolates originating from ILI and SARI patients that had no history of oseltamivir treatment prior to sampling. Among non-mutant viruses, 45 of 46 showed IC_50_ values ranging from 0. 06 to 0·37 nm (median IC_50_ 0·35 nm). In contrast, two mutant viruses had IC_50_ values of 127·91 nm (2011) and 125·02 nm (2013), approximately 200-fold higher than the reference wild-type virus A/Perth/265/2009 (IC_50_ 0·6 nm), around 18-fold lower than reference mutant virus A/Perth/265/2009 (IC_50_ 2179 nm), and 300-fold higher than median IC_50_ value (data not shown).

### The D222N mutation in relation to disease severity

A deduced aspartic acid to asparagine substitution observed at position 222 (D222N) of the HA1 protein was detected in a virus isolated from a 45-year-old male patient in November 2013. The patient had fever, cough, and dyspnea that progressed over 5 days leading to hospital admission. At the initial clinical assessment, the oxygen saturation (SaO_2_) was 92%, pulmonary infiltrates were present in both lungs, and the leukocyte count was 9300 cells/ml with 85% neutrophils. Despite oseltamivir and antibiotic treatment and intensive care, the patient's condition continued to deteriorate and he died 7 days after admission from multi-organ failure (data not shown).

## Discussion

Following WHO recommendations for the post-pandemic phase of influenza A/H1N1pdm09 virus transmission, we conducted routine epidemiological and virological surveillance of influenza through the National Influenza Surveillance System (NISS) and Severe Acute Respiratory Infection (SARI) surveillance to monitor important genetic, antigenic, and functional changes in circulating viruses. Our results for 2009–2013 reveal that influenza A/H1N1pdm09 virus has become established as a seasonal influenza subtype and continues to cocirculate with other influenza viruses (A/H3N2, B) (Figure1). The genetic and antigenic analysis undertaken may provide understanding of the evolution of influenza A/H1N1pdm09 in Vietnam. Phylogenetic analysis of the HA and NA genes indicated consistency of Vietnamese A/H1N1pdm09 viruses with those circulating worldwide, and differentiation of HA into six groups/subgroups and NA gene into seven groups/subgroups (Figures3 & 4).^7^,^5^,^19^ Comparing amino acid residues in the HA and NA proteins of A/California/07/2009 with those in Vietnamese isolates, we identified some significant substitutions such as P83S, S203T, and R223Q in HA protein or N248D in NA protein that were observed in all study isolates (Table1). Common mutations in HA and NA genes detected in Vietnamese isolates were similar to other isolates from Thailand, China, and Taiwan. These findings suggest significant cross-border viral transmission among neighboring countries (Figures 3 & 4). We found five isolates (8·2%) with lower HI titers compared to vaccine candidate strain (A/California/07/09), and four of these (4/5) were collected in 2013 (Table2). The A/H1N1pdm09 low reactors in HI assay have also been detected by the National Institute of Infectious Diseases, Tokyo, Japan (18%), and Victorian Infectious Diseases Reference Laboratory, Melbourne, Australia (8%) in 2013.^20^,^21^ The association of mutation N156K on the HA1 with both antigenic drift and virus receptor binding of A/H1N1pdm09 was determined in a ferret model^19^ and was confirmed by our study as all low-titer isolates possessed both the G155E and the N156K mutations (Table2). The increasing number of lower HI titer viruses in 2013 (4/5 viruses) and their concentration in the same cluster with other mutations among subgroup 6C viruses (Figure3) suggests some degree of antigenic drift of A/H1N1pdm09. However, the mutation G155E has been frequently observed in association with propagation in cell culture, while mutation N156K is more common in original specimens than isolates^19^; therefore, further direct sequencing of clinical samples will be required to assess the need to update vaccine virus strains.^19^,^22^

The D222G mutation in the HA of A/H1N1pdm/09 viruses was first reported in Norway in November 2009, raising a possible link between this mutation and greater virulence.^23^ The frequency of D222G-mutated viruses isolated only from severe or fatal cases was documented in Norway, USA, UK, and Spain.^23^–^26^ Our study reports a variant D222N virus from a fatal case in 2013. Similar to the D222G mutation, the variants D222A, D222E, and D222N also result in enhanced binding to receptor (α2-3) of epithelial cells in the lower respiratory tract, whereas wild-type seasonal influenza strains bind preferentially to the α2-6 receptor of the upper airway cells.^26^ This finding was reported from Spain in 2010, but it is unclear what implications this might have in terms of severity.^27^ However, in early 2011, the D222N substitution was found in three cases in the United States and may correlate with severe clinical manifestations, similar to D222G.^28^ Our result provides additional information about the variant D222N in association with enhanced virulence of influenza A/H1N1pdm09 virus. Two oseltamivir-resistant A/H1N1pdm09 isolates in 2011 and 2013 were detected by neuraminidase inhibition (NAI) test and Sanger sequence NA gene (H275Y); in total, 11 cases of Vietnamese oseltamivir-resistant A/H1N1pdm09 isolates have been identified between 2009 and 2013.^10^,^29^

After 4-year circulation in the post-pandemic period, influenza A/H1N1pdm09 has taken its place alongside influenza A/H3N2 and B as a seasonal influenza strain in Vietnam and worldwide. Continued vigilance is needed to monitor viral evolution that might enhance resistance to oseltamivir and/or pathogenicity or affect vaccine efficacy. Our results provide virological data of circulating influenza A/H1N1pdm09 in Vietnam that will inform development of influenza vaccine strategies for Vietnamese people, as well as contribute to strengthening the Global Influenza Surveillance and Response System (GISRS).
